# Optimal Strategy for Antiplatelet Therapy After Coronary Drug-Eluting Stent Implantation in High-Risk “TWILIGHT-like” Patients With Diabetes Mellitus

**DOI:** 10.3389/fcvm.2020.586491

**Published:** 2020-11-27

**Authors:** Hao-Yu Wang, Zhong-Xing Cai, Dong Yin, Wei-Hua Song, Lei Feng, Run-Lin Gao, Yue-Jin Yang, Ke-Fei Dou

**Affiliations:** ^1^Department of Cardiology, Center for Coronary Heart Disease, Fuwai Hospital, National Center for Cardiovascular Diseases, Chinese Academy of Medical Sciences and Peking Union Medical College, Beijing, China; ^2^State Key Laboratory of Cardiovascular Disease, Beijing, China; ^3^National Clinical Research Center for Cardiovascular Diseases, Beijing, China

**Keywords:** diabetes mellitus, dual antiplatelet therapy, high-risk patients, bleeding, thrombosis, drug-eluting stents, percutaneous coronary intervention

## Abstract

**Background:** Patients with diabetes mellitus (DM) are known to be at high-risk for both ischemic and bleeding complications post-percutaneous coronary intervention (PCI). The ischemic benefit vs. bleeding risk associated with extended dual antiplatelet therapy (DAPT) in high-risk “TWILIGHT-like” patients with diabetes mellitus after PCI has not been established.

**Methods:** All consecutive high-risk patients fulfilling the “TWILIGHT-like” criteria undergoing PCI from January 2013 through December 2013 were identified from the prospective Fuwai PCI Registry. High-risk “TWILIGHT-like” patients were defined by at least one clinical and one angiographic feature based on the TWILIGHT trial selection criteria. The present analysis evaluated 3,425 diabetic patients with concomitant high-risk angiographic features who were event-free at 1 year after PCI. Median follow-up was 2.4 years. The primary effectiveness endpoint was a composite of death, myocardial infarction, or stroke (termed major adverse cardiac and cerebrovascular events), and primary safety endpoint was clinically relevant bleeding according to the Bleeding Academic Research Consortium types 2, 3, or 5.

**Results:** On inverse probability of treatment weighting (IPTW) analysis, prolonged-term (>1-year) DAPT with aspirin and clopidogrel decreased the risk of primary effectiveness endpoint compared with shorter ( ≤ 1-year) DAPT [1.8 vs. 4.3%; hazard ratio (HR)_IPTW_: 0.381; 95% confidence interval (CI): 0.252–0.576; *P* < 0.001] and reduced cardiovascular death [0.1% vs. 1.8%; HR_IPTW_: 0.056 (0.016–0.193)]. Prolonged DAPT was also associated with a reduced risk of definite/probable stent thrombosis [0.2 vs. 0.7%; HR_IPTW_: 0.258 (0.083–0.802)] and non-significantly lower rate of myocardial infarction [0.5 vs. 0.8%; HR_IPTW_: 0.676 (0.275–1.661)]. There was no significant difference between groups in clinically relevant bleeding [1.1 vs. 1.1%; HR_IPTW_: 1.078 (0.519–2.241); *P* = 0.840). Similar results were observed in multivariable Cox proportional hazards regression model.

**Conclusion:** Among high-risk PCI patients with diabetes mellitus without an adverse event through 1 year, extending DAPT >1-year significantly reduced the risk of major adverse cardiac and cerebrovascular events without an increase in clinically relevant bleeding, suggesting that such high-risk diabetic patients may be good candidates for long-term DAPT.

## Introduction

The addition of a P2Y_12_ inhibitor on a background of aspirin therapy is standard of care after percutaneous coronary intervention (PCI) for the prevention of ischemic complications (1, 2). The optimal duration of DAPT has remained controversial, owing to refinements in DES technologies and the advent of potent P2Y_12_ antagonist (3). Multiple trials evaluating whether treatment with DAPT over 12 months enabled reduction of the risk of either stent thrombosis (ST) or atherothrombotic complications related to sites outside the stented segment in certain patient populations remained disputed (4–8), in which some have confirmed its benefit value (4, 5), and some have not (6–8). However, concerns of adverse events with prolonged DAPT, mainly mediated through bleeding, have promoted randomized controlled trials (RCTs) exploring the effect of shorter DAPT duration in subjects with low-ischemic risk (9). Therefore, to clarify patients who could potentially benefit from a longer duration of DAPT with adequate thrombotic protection without a trade-off in major bleeding, clinicians are warranted to balance the estimated risks of recurrent ischemic and bleeding events based on a mindful evaluation of patient's risk profile that include clinical and procedural variables.

Under this scenario, the TWILIGHT study enrolled a high-risk population with at least one clinical [e.g., diabetes mellitus (DM)] and one angiographic (e.g., complex coronary artery disease) features who are at high risk of ischemic or bleeding events after PCI (10, 11). There is recognition that patients with DM represent a unique population as this metabolic disorder incur a significantly higher risk of ischemic and bleeding complications (12–14). Part of the effect of DM may be explained by the contribution of concomitant comorbidities, procedural complexity, and insulin resistance and hyperglycemia to a prothrombotic state, resulting in endothelial dysfunction, coagulative activation, and platelet hyper-reactivity (13–15). Importantly, the prevalence of DM continues to grow during the past 10 years (16), a fact that is also reflected in the PCI trials wherein DM is a commonly occurring major risk amplifier in patients with coronary artery disease (CAD). These observations highlight the importance of selecting appropriate strategy for antiplatelet therapy in patients with DM after PCI. When DM is coexistent with high-risk angiographic factors, more intense and longer duration antiplatelet therapy could be of more benefit for secondary prevention of atherothrombotic recurrences in these high-risk patients. Given that DM was a key clinical inclusion criterion in “TWILIGHT-like” patients and the role of DM as an important corelate of thrombotic events, whether patients with DM and concomitant high-risk angiographic feature would benefit from long-term DAPT remains uncertain. To answer this question, we aimed to assess the efficacy and safety of extended (>1-year) duration DAPT vs. shorter DAPT ( ≤ 1-year) on clinical outcomes in a large and contemporary cohort of high-risk “TWILIGHT-like” diabetic patients undergoing PCI.

## Methods

### Study Population

**Figure 1:**
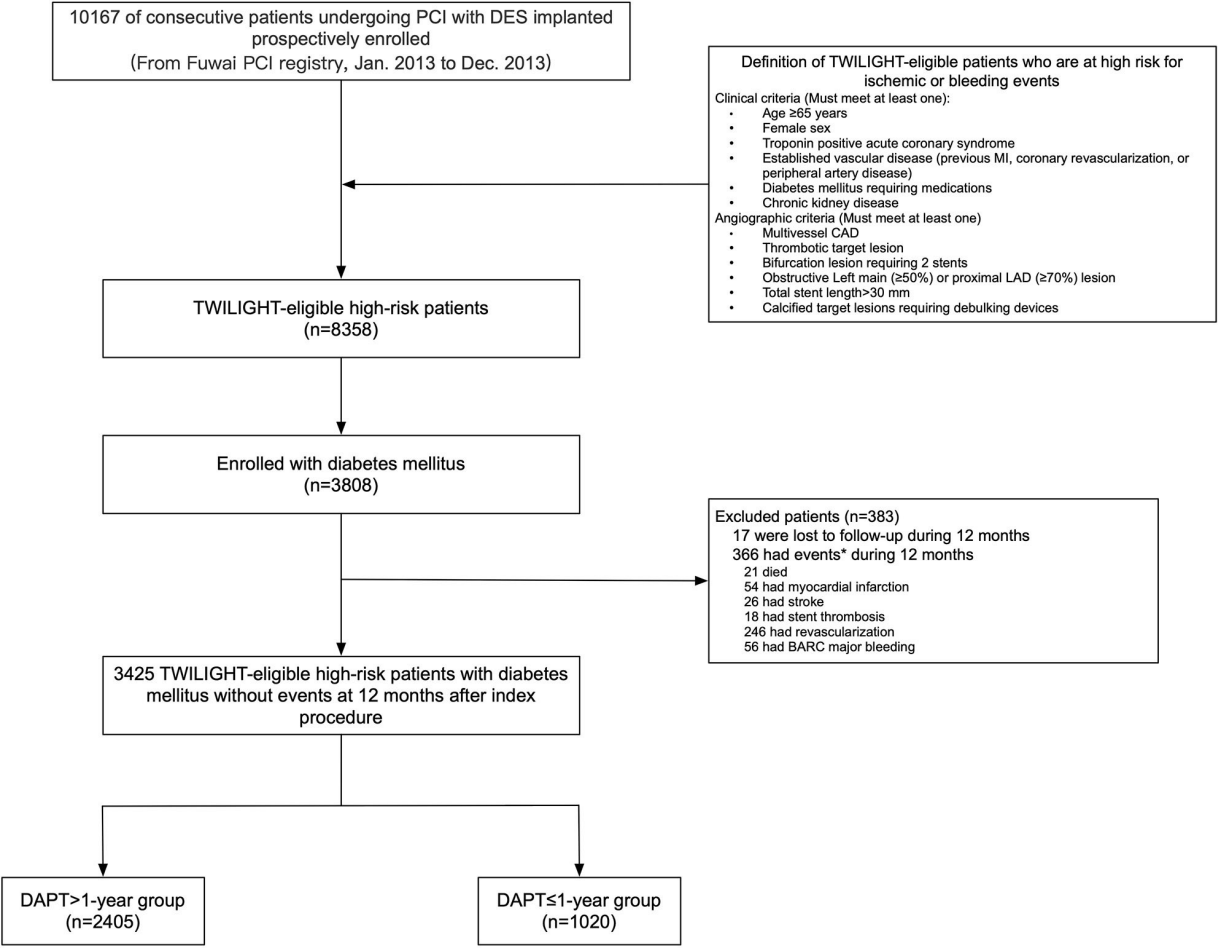
Study cohort. *Subjects may have >1 event. BARC, Bleeding Academic Research Consortium; CAD, coronary artery disease; DES, drug-eluting stent; DAPT, dual antiplatelet therapy; LAD, left anterior descending; MI, myocardial infarction; PCI, percutaneous coronary intervention.

The Fuwai registry was a large single-center, prospective, observational study that consecutively enrolled 10,167 patients with CAD treated with PCI with implantation of at least one drug-eluting stents (DES) between January 2013 and December 2013 in Fu Wai Hospital, National Center for Cardiovascular Diseases, Beijing, China. This prospective PCI registry complied with the provisions of the Declaration of Helsinki and was approved by the institutional ethics committee at Fuwai Hospital, Beijing, China. All eligible patients signed a written informed consent for participation in this registry. For purposes of the present analysis, we used the TWILIGHT trial selection criteria to identify the TWILIGHT-like population (10, 11). Then, 8,358 consecutive high-risk “TWILIGHT-like” patients fulfilling the TWILIGHT inclusion criteria were identified, who had at least one of six procedural characteristics and at least one of six clinical characteristics in terms of their association with high ischemic or bleeding risk. The clinical criteria for high risk were aged at least 65 years, women, troponin-positive acute coronary syndrome (ACS), chronic kidney disease (CKD; estimated glomerular filtration rate of <60 ml/min), DM requiring medication, documented atherosclerosis [prior myocardial infarction (MI) or peripheral arterial disease (PAD)] or revascularization. Angiographic criteria included multivessel CAD, thrombotic lesions, total stent length >30 mm, obstructive left main (LM)/proximal left anterior descending (LAD) lesion, bifurcation lesions with two-stent strategy, and calcified target lesions using debulking devices. For the present analysis, patients with DM (*n* = 3,808) were defined as patients who had been treated with oral hypoglycemic agents or insulin or those with hemoglobin A1c (HbA1c) ≥6.5% at baseline, based on the current guidelines (17). For purposes of the present analysis, we included patients who have completed the first year after PCI and were free of all-cause mortality, MI, stroke, repeat revascularization, definite or probable stent thrombosis (ST), or Bleeding Academic Research Consortium (BARC) major bleeding at the 1 year follow-up. Finally, 3,425 high-risk “TWILIGHT-like” patients with DM qualified for the present analysis and were classified into two groups (DAPT > 1-year vs. DAPT ≤ 1-year) (Figure 1). We analyzed the data using a 1-year landmark and evaluated outcomes from the landmark time point stratified by the duration of DAPT.

### PCI Procedures, Data Collection, and Follow-Up

PCI was done according to the standard techniques at the discretion of the treating physician (Methods in the Supplementary File) (18). Baseline and procedural characteristics, findings of coronary angiography, clinical diagnosis, and clinical events were systematically obtained using standardized forms at the time of index hospitalization for PCI. Attending physicians followed patients at 30 days, 6 months, 12 months after discharge, and annually thereafter. Patients were followed up through outpatient clinical visit, telephone calls, or medical records review. Dedicated independent clinical research coordinators collected and inputted all data obtained during the follow-up visits. At these visits, the data pertaining to patient clinical status, angiographic and procedural characteristics, and in-hospital and follow-up adverse events, were recorded. At follow-up, antiplatelet medication (e.g., aspirin and P2Y_12_ inhibitor) status was also checked by dedicated questionnaires.

### Endpoint Definitions

The primary efficacy outcome of major adverse cardiac and cerebrovascular events (MACCE) was a composite of death from any cause, MI, and stroke. The safety endpoint was the incidence of clinically relevant bleeding defined by the BARC types 2, 3, or 5. The net clinical benefit outcome was defined as death from any cause, MI, stroke, or clinically relevant bleeding. Other endpoints analyzed were the individual components of the primary efficacy outcome, the incidence of cardiovascular death, stent thrombosis (definite or probable), and ischemic stroke.

All in-hospital and post-discharge events with relevant medical records were monitored and adjudicated by an independent clinical events committee whose members were unaware of this study. Cardiovascular death was defined as a death for which a definite non-cardiovascular cause (e.g., cancer) has not been identified. Uncertain causes of deaths are presumed to be cardiovascular unless proven otherwise. Based on the Third Universal Definition of MI, MI was defined as a rise in cardiac biomarkers (creatine kinase MB fraction or cardiac troponin) above the 99th percentile of the upper normal limit, in conjunction with symptoms of ischemia, electrocardiographic changes, or abnormal imaging findings (19). Target vessel-related MI was one related to the target vessel, or the MI could not be clearly related to another vessel. Revascularization and ST (definite or probable) were adjudicated according to the definitions provided by the Academic Research Consortium (20). Stroke was defined as any non-convulsive focal or global neurological deficit of abrupt onset caused by an ischemic or hemorrhagic event, with residual symptoms lasting >24 h or leading to death. The definition of bleeding events was categorized using the BARC criteria (21).

### Statistical Analysis

Data for continuous variables are summarized as mean ± SD, and categorical variables are presented as number and corresponding percentages. Variables were compared between patients with and without DAPT >1-year using the chi-square or Fisher exact (for categorical variables) or Student t (for continuous variables) test. The cumulative incidence of clinical outcomes was calculated using the Kaplan–Meier method. Kaplan–Meier survival curves were compared using the log-rank test. The prognostic impact of extended DAPT beyond 1 year was tested in Cox proportional hazard regression analyses. Known risk factors of the study endpoints were included as covariates: covariates for ischemic endpoints included age, sex, current smoker, ACS, CKD, hypertension, PAD, prior MI, prior PCI or coronary artery bypass graft (CABG), left ventricular ejection fraction (LVEF), DES type, multivessel disease, treated lesion in the LM or LAD artery, total lesion length, and total stents numbers. For clinically relevant bleeding, we included following variables in the adjusted models: age, sex, body mass index (BMI), CKD, ACS, history of major bleeding, and anemia; and for net clinical benefit outcome, age, sex, BMI, current smoker, CKD, ACS, hypertension, PAD, prior MI, prior PCI or CABG, LVEF, DES type, history of major bleeding, anemia, multivessel disease, treated lesion in the LM or LAD artery, total lesion length, and total stent numbers were entered into a multivariate model. As a sensitivity analysis, the Fine–Gray subdistribution hazards model was used to account for the competing risk of non-cardiovascular death when assessing MACCE (cardiovascular death, MI, or stroke)-free survival of DAPT >1-year vs. DAPT ≤ 1-year.

To minimize the possibility of biased effect estimates in observational studies, weighted Cox proportional hazards regression models using inverse probability of treatment weighting (IPTW) was used to adjust for differences in the baseline characteristics for drawing inferences about the relative effectiveness of DAPT >1-year vs. DAPT ≤ 1-year. The propensity score (PS) has been developed using a non-parsimonious multivariable logistic regression model and considering DAPT duration (DAPT >1-year vs. DAPT ≤ 1-year) as dependent variable. IPTW techniques involve assigning each patient a weight (1–p)/(1–PS) if a control, or weight p/PS if a treated patient, where p is the probability of treatment without any covariate, and PS is the value of the PS for that patient. After IPTW adjustment, weighted standardized mean differences (SMD) of each covariate with values <0.10 indicated an acceptable balance. Covariates used for the propensity score model are shown in the Supplementary File. We also performed a Cox regression analysis with interaction testing to determine whether the effect of DAPT duration on the primary efficacy endpoint, on the primary safety endpoint, and on net clinical benefit was consistent across the number of TWILIGHT inclusion criteria fulfilled (1–3, 4–5, or 6–9 characteristics) and important subgroups (age ≥ 65, sex, CKD, BMI, current smoker, clinical presentation, STEMI, history of PCI, multivessel disease, and total stent length > 30 mm). A two-side value of *P* < 0.05 was considered statistically significant. All analyses were performed on SPSS version 24.0 (SPSS Inc., Chicago, IL, USA) and R version 3.6.0 (R Foundation for Statistical Computing, Vienna, Austria).

## Results

### Baseline, Angiographic, and Procedural Data

Among 3,425 high-risk “TWILIGHT-like” patients with DM who were free of ischemic or hemorrhagic events occurring within the first year, 2,405 (70.2%) remained on DAPT >1-year, for a mean duration of 671 days (SE: 3.41), while 1,020 patients had DAPT discontinuation within 1 year with a mean duration of 350 days (SE: 1.83). A total of 31.9% of patients with an age of at least 65 years, 26.7% were women, 58.9% had ACS, 41.5% had established vascular disease, 5.2% had CKD, 86.3% had multivessel CAD, 63.8% had total stent length>30 mm, 3.9% had thrombotic target lesion, 4.4% had bifurcation lesion treated with two stents, 42.5% had LM (≥50%) or proximal LAD (≥70%) lesion, and 0.6% had severely calcified lesion (requiring a rotablator system). Table 1 lists the baseline data of the DM cohort, which were well-balanced with no statistically significant differences between the two groups, except for HbA1c, PAD, ACS, and statin use. In terms of procedural characteristics, extended DAPT had a higher prevalence of multivessel CAD (Table 2). The clinical and angiographic characteristics stratified by DAPT duration (>1-year vs. ≤ 1-year DAPT) were well-balanced after IPTW with all standardized differences <10% (Supplementary Table 1).

**Table 1:** Baseline clinical characteristics in high-risk patients with diabetes mellitus stratified by dual antiplatelet therapy (DAPT) duration.

	**DAPT >1-year (*n* = 2,405)**	**DAPT ≤1-year (*n* = 1,020)**	***P*-value**
Age, years	59.68 ± 9.88	59.45 ± 9.75	0.523
Male	1,745 (72.6)	766 (75.1)	0.124
Body mass index, kg/m^2^	26.30 ± 3.16	26.26 ± 3.20	0.733
Hypertension	1,689 (70.2)	702 (68.8)	0.413
Hyperlipidemia	1,764 (73.3)	723 (70.9)	0.139
Chronic kidney disease	135 (5.6)	42 (4.1)	0.071
Current smoker	1,309 (54.4)	578 (56.7)	0.228
Peripheral artery disease	97 (4.0)	25 (2.5)	0.022
Prior MI	556 (23.1)	209 (20.5)	0.091
Prior PCI	664 (27.6)	270 (26.5)	0.494
Prior CABG	130 (5.4)	53 (5.2)	0.803
Prior stroke	302 (12.6)	142 (13.9)	0.277
LVEF, %	62.43 ± 7.56	62.67 ± 7.53	0.265
Indication for PCI			0.008
Stable CAD	1,023 (42.5)	384 (37.6)	
ACS	1,382 (57.5)	636 (62.4)	
UA/NSTEMI	1,115 (46.4)	502 (49.2)	0.126
STEMI	267 (11.1)	134 (13.1)	0.090
Hemoglobin, g/dL	14.19 ± 1.58	14.21 ± 1.52	0.746
Platelet count, 10^3^/dL	204.37 ± 55.98	205.87 ± 57.05	0.478
White blood cell count, 10^3^/mL	6.86 ± 1.69	6.86 ± 1.62	0.891
HbA1c, %	7.65 ± 1.34	7.49 ± 1.32	0.001
PARIS thrombotic risk score	3.33 ± 1.84	3.32 ± 1.73	0.865
PARIS bleeding risk score	3.79 ± 2.08	3.68 ± 1.95	0.137
PRECISE-DAPT score	11.58 ± 8.65	11.15 ± 8.67	0.189
DAPT score	2.13 ± 1.30	2.15 ± 1.28	0.739
Medication			
Aspirin	2,379 (98.9)	1,007 (98.7)	0.626
Clopidogrel	2,373 (98.7)	1,009 (98.9)	0.544
Beta-blocker	2,222 (92.4)	937 (91.9)	0.597
Calcium channel blockers	1,220 (50.7)	520 (51.0)	0.892
Statin	2,309 (96.0)	962 (94.3)	0.029
Antidiabetic drugs at baseline			
OADs	976 (40.6)	443 (43.4)	0.122
Insulin	613 (25.5)	247 (24.2)	0.432

*Values are n (%) or mean ± SD. ACEI, angiotensin-converting enzyme inhibitors; ACS, acute coronary syndrome; ARB, angiotensin receptor blockers; CABG, coronary artery bypass grafting; DAPT, dual antiplatelet therapy; HbA1c, glycosylated hemoglobin; LVEF, left ventricular ejection fraction; MI, myocardial infarction; NSTEMI, non-ST-segment elevation myocardial infarction; OADs, oral antidiabetic drugs; PCI, percutaneous coronary intervention; STEMI, ST-segment elevation myocardial infarction; UA, unstable angina*.

**Table 2:** Procedural characteristics in high-risk patients with diabetes mellitus stratified by DAPT duration.

	**DAPT >1-year (*n* = 2,405)**	**DAPT ≤1-year (*n* = 1,020)**	***P*-value**
Multivessel CAD	2,099 (87.3)	856 (83.9)	0.009
Target vessel			
Left anterior descending artery	2,131 (88.6)	898 (88.0)	0.635
Left circumflex artery	497 (20.7)	204 (20.0)	0.659
Right coronary artery	522 (21.7)	221 (21.7)	0.980
Left main coronary artery	84 (3.5)	28 (2.7)	0.261
Bypass graft	8 (0.3)	2 (0.2)	0.498
Total lesion length, mm	42.22 ± 27.81	40.46 ± 26.09	0.084
Number of vessels treated	1.31 ± 0.52	1.30 ± 0.51	0.467
Number of lesions treated			0.156
1	1,499 (62.3)	634 (62.2)	
2	688 (28.6)	312 (30.6)	
≥3	218 (9.1)	74 (7.3)	
Number of stents implanted	2.06 ± 1.13	2.00 ± 1.04	0.138
≥3 stents implanted	655 (27.2)	262 (25.7)	0.349
Total stent length, mm	45.94 ± 28.43	44.45 ± 26.00	0.151
Total stent length >30 mm	1,532 (63.7)	652 (63.9)	0.902
Mean stent diameter, mm	2.96 ± 0.54	2.98 ± 0.54	0.318
Target lesion morphology			
Bifurcation	400 (16.6)	152 (14.9)	0.208
Chronic total occlusion	217 (9.0)	78 (7.6)	0.189
In-stent restenosis	134 (5.6)	52 (5.1)	0.576
Severe calcification	97 (4.0)	34 (3.3)	0.329
Thrombotic lesion	89 (3.7)	44 (4.3)	0.396
Type B2 or C lesion	1,938 (80.6)	814 (79.8)	0.600
SYNTAX score	12.51 ± 8.15	12.15 ± 8.16	0.240
Vascular access site			0.471
Radial	2,169 (90.2)	928 (91.0)	
Femoral	236 (9.8)	92 (9.0)	
Intravascular ultrasound use	139 (5.8)	55 (5.4)	0.654
Glycoprotein IIb/IIIa use	374 (15.6)	177 (17.4)	0.189
DES type			0.971
DES, first-generation	258 (10.7)	109 (10.7)	
DES, second-generation	2,147 (89.3)	911 (89.3)	

*Values are n (%) or mean ± SD. CAD, coronary artery disease; DES, drug-eluting stent*.

### Clinical Outcomes

**Figure 2:**
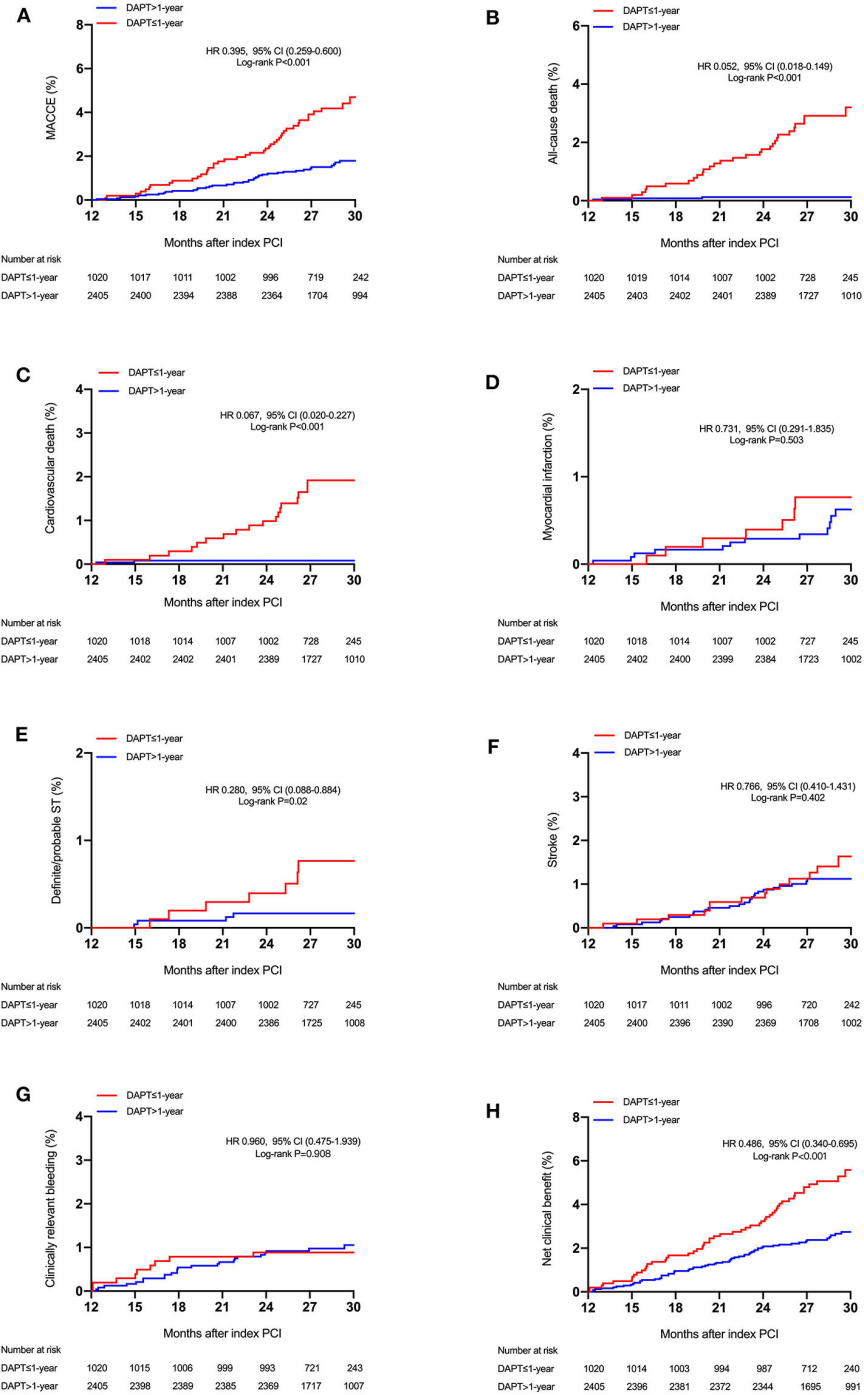
Cumulative incidence of the study outcomes stratified by DAPT duration. Time-to-event curves for **(A)** major adverse cardiovascular or cerebrovascular events (MACCE) (all-cause mortality, myocardial infarction, or stroke), **(B)** all-cause mortality, **(C)** cardiovascular mortality, **(D)** myocardial infarction, **(E)** stent thrombosis (definite or probable), **(F)** stroke, **(G)** clinically relevant bleeding, and **(H)** net clinical benefit, according to the duration of DAPT (≤ 1-year vs. >1-year DAPT). MACCE indicates major adverse cardiac and cerebrovascular events.

During a median follow-up of 2.4 years (interquartile range, 2.2–2.6 years), a total of 88 MACCEs, including 34 all-cause mortality, 20 MI, and 44 strokes were recorded. At least 2 years of follow-up data were available for 3,406 patients (99.4%) after PCI. Patients lost to follow-up were censored at the last known follow-up date. Patients with >1-year DAPT had lower unadjusted rates of MACCE (1.8 vs. 4.3%; *P* < 0.001), all-cause and cardiovascular (0.1 vs. 1.8%; *P* < 0.001) mortality, and definite/probable ST (0.2 vs. 0.7%; *P* = 0.02) but similar rates of MI, stroke, and clinically relevant bleeding (1.1 vs. 1.1%; *P* = 0.908) compared with ≤ 1-year DAPT group (Table 3 and Figure 2). As expected, the unadjusted rates of net clinical benefit (2.8 vs. 5.4%; *P* < 0.001) were higher in shorter (≤ 1-year) DAPT compared with longer (>1-year) DAPT. By multivariable Cox regression analysis adjusting principally for clinical covariates, prolonged-term (>1-year) DAPT was associated with a reduced risk of MACCE compared with abbreviated-term (<1-year) DAPT [adjusted hazard ratio (HR): 0.371; 95% CI, 0.244–0.566; *P* < 0.001). In comparison with discontinuation of DAPT within 1 year after PCI, continued DAPT significantly reduced the risk of cardiovascular (adjusted HR: 0.062; 95% CI, 0.018–0.212; *P* < 0.001) and all-cause mortality (adjusted HR: 0.049; 95% CI, 0.017–0.141; *P* < 0.001), as well as definite/probable ST (adjusted HR: 0.255; 95% CI, 0.079–0.821; *P* = 0.022). Compared with ≤ 1-year DAPT, patients treated with DAPT >1-year had trends toward less MI and stroke, but without significant between-group differences. When we examined safety outcomes, extended-term DAPT did not appear to increase the risk of clinically relevant bleeding (adjusted HR: 1.038; 95% CI, 0.501–2.151; *P* = 0.921). In an attempt to define net clinical benefit, the risk of the net clinical benefit outcome, comprising all-cause death, MI, stroke, or clinically relevant bleeding was lower with >1-year DAPT compared with ≤ 1-year DAPT (adjusted HR: 0.467; 95% CI, 0.325–0.670; *P* < 0.001).

**Table 3:** Adverse clinical events in high-risk diabetic patients according to DAPT duration.

	**DAPT >1-year (*n* = 2,405)**	**DAPT ≤1-year (*n* = 1,020)**	**Univariate analysis**		Multivariable analysis^*^		**IPTW analysis**	
			**HR (95% CI)**	***P*-value**	**HR (95% CI)**	***P*-value**	**HR (95% CI)**	***P*-value**
Major adverse cardiac and cerebrovascular events	44 (1.8%)	44 (4.3%)	0.395 (0.259–0.600)	<0.001	0.371 (0.244–0.566)	<0.001	0.381 (0.252–0.576)	<0.001
CV death, myocardial infarction, or ischemic stroke	39 (1.6%)	33 (3.2%)	0.470 (0.295–0.748)	0.001	0.440 (0.276–0.702)	0.001	0.440 (0.280–0.693)	<0.001
All-cause death	4 (0.2%)	30 (2.9%)	0.052 (0.018–0.149)	<0.001	0.049 (0.017–0.141)	<0.001	0.047 (0.016–0.136)	<0.001
CV death	3 (0.1%)	18 (1.8%)	0.067 (0.020–0.227)	<0.001	0.062 (0.018–0.212)	<0.001	0.056 (0.016–0.193)	<0.001
Myocardial infarction	13 (0.5%)	7 (0.8%)	0.731 (0.291–1.835)	0.505	0.694 (0.275–1.753)	0.440	0.676 (0.275–1.661)	0.394
Stroke	29 (1.2%)	15 (1.5%)	0.766 (0.410–1.431)	0.404	0.725 (0.386–1.360)	0.316	0.784 (0.419–1.466)	0.446
Ischemic stroke	25 (1.0%)	14 (1.4%)	0.713 (0.370–1.374)	0.312	0.676 (0.349–1.311)	0.247	0.726 (0.378–1.393)	0.335
Definite/probable stent thrombosis	5 (0.2%)	7 (0.7%)	0.280 (0.088–0.884)	0.030	0.255 (0.079–0.821)	0.022	0.258 (0.083–0.802)	0.019
Clinically relevant bleeding	27 (1.1%)	11 (1.1%)	0.960 (0.475–1.939)	0.908	1.038 (0.501–2.151)	0.921	1.078 (0.519–2.241)	0.840
Net clinical benefit	68 (2.8%)	55 (5.4%)	0.486 (0.340–0.695)	<0.001	0.467 (0.325–0.670)	<0.001	0.485 (0.340–0.691)	<0.001

*Data presented as number of events (%)*.

* *The candidate covariates considered for inclusion in the model for ischemic outcomes were age, sex, current smoker, hypertension, chronic kidney disease, acute coronary syndrome, left ventricular ejection fraction, peripheral artery disease, prior MI, prior PCI or CABG, DES type, multivessel CAD, treated lesion in the left main or left anterior descending artery, total lesion length, and total stents numbers. The candidate covariates considered for inclusion in the model for clinically relevant bleeding were age, sex, body mass index, chronic kidney disease, acute coronary syndrome, history of major bleeding, and anemia. The candidate covariates considered for inclusion in the model for net clinical benefit were age, sex, body mass index, current smoker, hypertension, chronic kidney disease, acute coronary syndrome, left ventricular ejection fraction, peripheral artery disease, prior MI, prior PCI or CABG, DES type, multivessel CAD, treated lesion in the left main or left anterior descending artery, total lesion length, total stents numbers, history of bleeding, and anemia*.

*Anemia is defined as hemoglobin <12 g/dl for male and <11 g/dl for female*.

*CI, confidence interval; CV, cardiovascular; DAPT, dual antiplatelet therapy; HR, hazard ratio; IPTW, inverse probability of treatment weighting; Other abbreviations as in Tables 1, 2*.

These results were consistently observed after the weighted Cox proportional hazards regression models using the IPTW method (Table 3). Extended duration DAPT led to a marked reduction in the risk of MACCE [HR_IPTW_: 0.381 (95% CI: 0.252–0.576), *P* < 0.001] when compared with short duration treatment, with directional consistency for cardiovascular death [HR_IPTW_: 0.056 (95% CI: 0.016–0.193)] and definite/probable ST [HR_IPTW_: 0.258 (95% CI, 0.083–0.802)]. There was no increase in clinically relevant bleeding [HR_IPTW_: 1.622 (95% CI, 0.631–4.169)] risk in the longer than 1-year DAPT group compared with the shorter DAPT group. As a result, a net clinical benefit of 51% was seen in patients treated with long-term DAPT [HR_IPTW_: 0.485 (95% CI, 0.340–0.691)].

### Sensitivity and Subgroup Analyses

A sensitivity analysis performed to assess the treatment effect of DAPT >1-year vs. DAPT ≤ 1-year was consistent after accounting for the competing risk of death. In both unadjusted and fully adjusted Fine and Gray competing risk models, extended-term (>1-year) DAPT was associated with lower rates of MACCE (a composite of cardiovascular death, MI, and stroke) compared with DAPT discontinuation within 1 year when adjusted for the competing risk of non-cardiovascular death and baseline risk factors [unadjusted subdistribution hazard ratio (sHR): 0.536; 95% CI, 0.340–0.846; *P* = 0.007; adjusted sHR: 0.517; 95% CI, 0.327–0.817; *P* = 0.005; Supplementary Figure 1].

**Figure 3:**
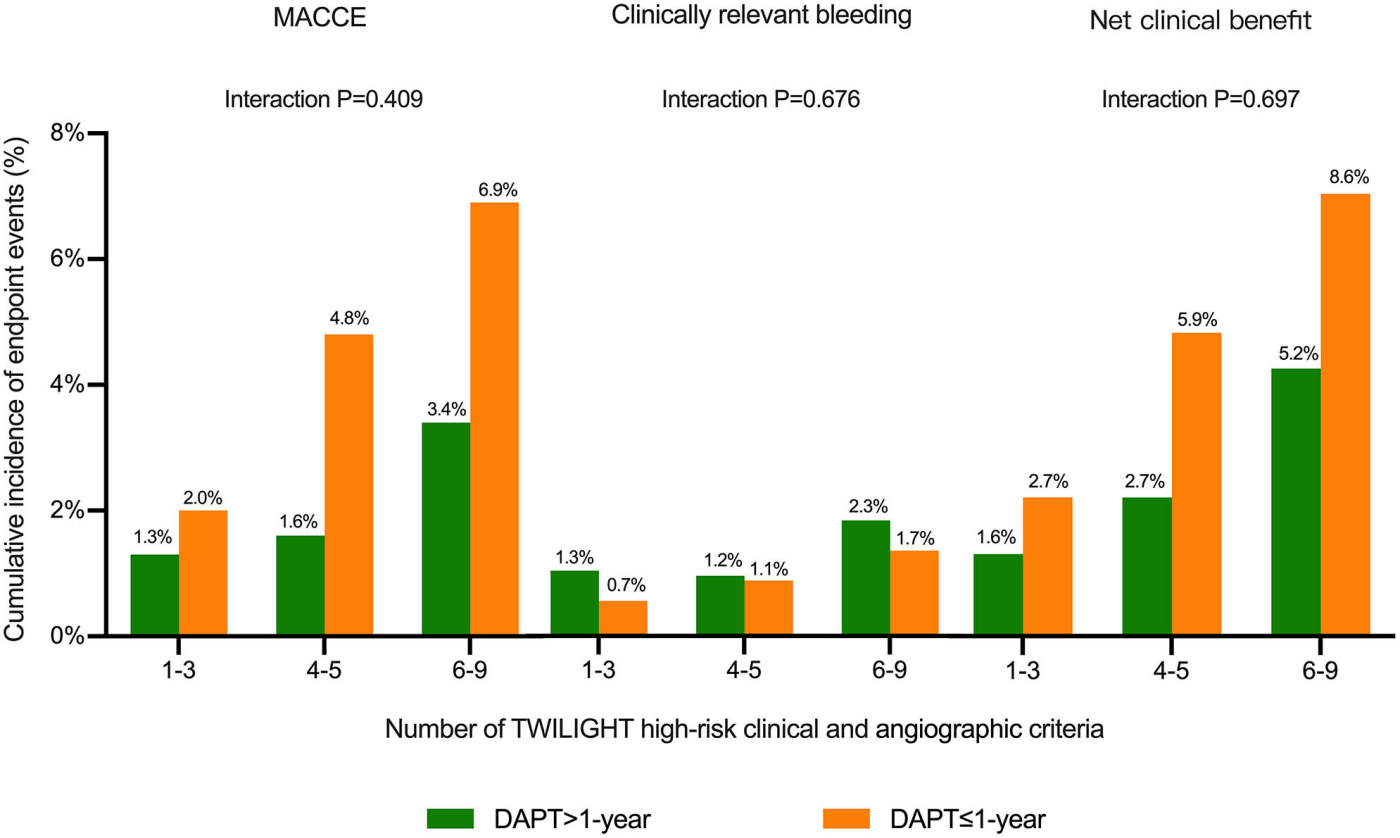
Cumulative incidence of endpoint events stratified by the number of TWILIGHT inclusion criteria fulfilled and duration of DAPT in patients with diabetes mellitus (DM). Outcomes were analyzed comparing DAPT >1-year vs. DAPT ≤ 1-year among subgroups of subjects 1–3 (*n* = 980), 4–5 (*n* = 1,827), or 6–9 (*n* = 618) TWILIGHT inclusion criteria to assess whether anti-ischemic effects of DAPT duration differed depending on the number of TWILIGHT inclusion features fulfilled in patients with DM. The treatment effects of extended DAPT over 1 year were consistent for the outcomes of MACCE, clinically relevant bleeding, and net clinical benefit outcome independent of the number of high-risk enrichment features fulfilled.

**Figure 4:**
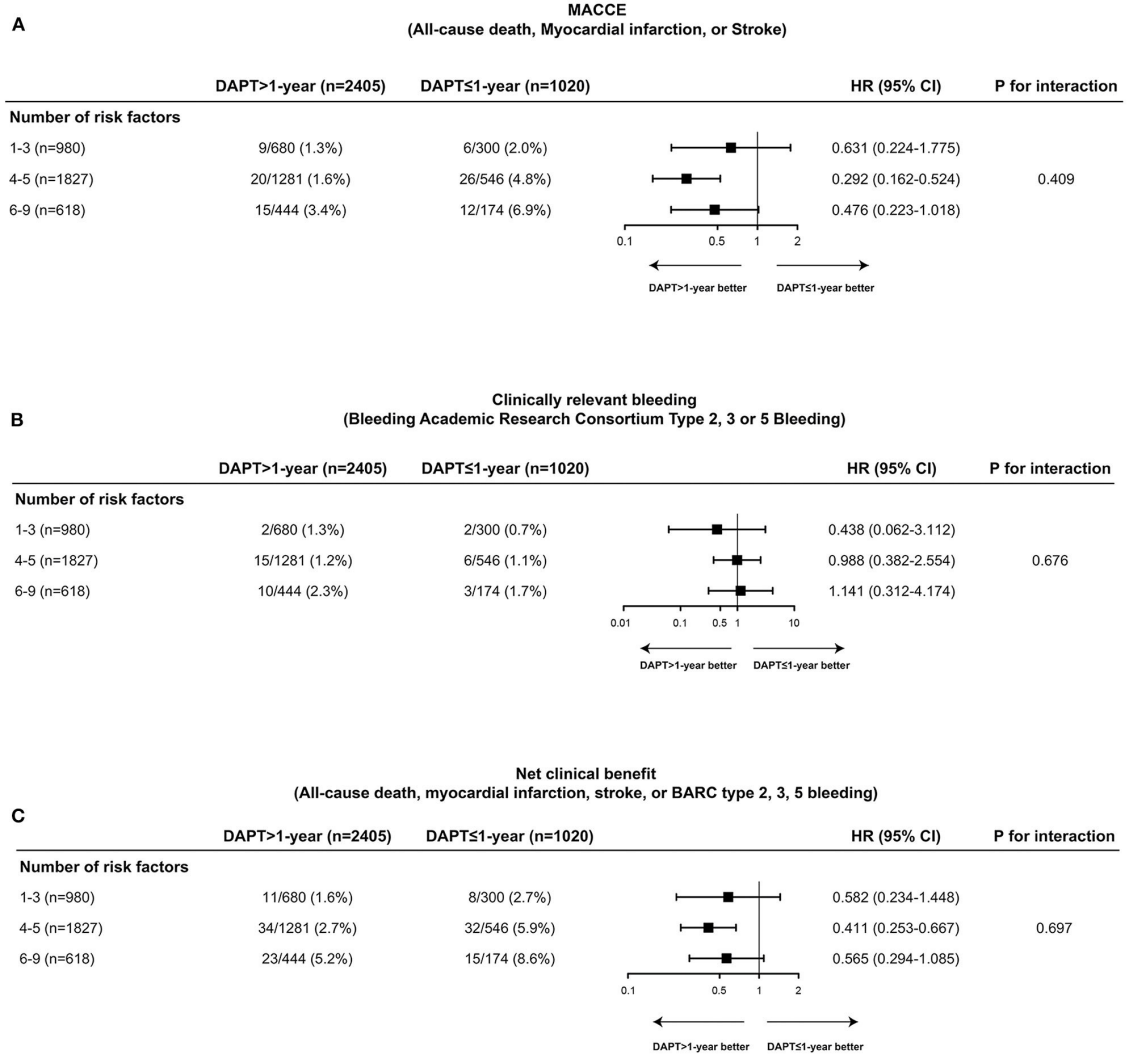
Comparative unadjusted hazard ratios of MACCE **(A)**, clinically relevant bleeding **(B)**, and net clinical benefit outcome **(C)** according to duration of DAPT stratified by number of TWILIGHT inclusion criteria fulfilled in patients with DM. The effect of DAPT >1-year vs. DAPT ≤ 1-year for MACCE, clinically relevant bleeding, and net clinical benefit outcome was consistent across patients with 1–3 (*n* = 980), 4–5 (*n* = 1,827), or 6–9 (*n* = 618) high-risk clinical and angiographic fulfilled in patients with DM. CI, confidence interval; HR, hazard ratio; MACCE, major adverse cardiovascular or cerebrovascular events.

The cumulative incidences of endpoint events were stratified by DAPT duration (≤ 1-year vs. >1-year DAPT), and the number of TWILIGHT inclusion criteria were fulfilled [1–3 (*n* = 980), 4–5 (*n* = 1,827), or 6–9 (*n* = 618) high-risk factors]. The risk differences between the two groups with respect to the primary efficacy and safety endpoints and the composite net clinical benefit outcome were independent of the progressive number of high-risk clinical and angiographic criteria fulfilled, with no significant treatment interactions (all P for interaction > 0.05) (Figures 3, 4).

**Figure 5:**
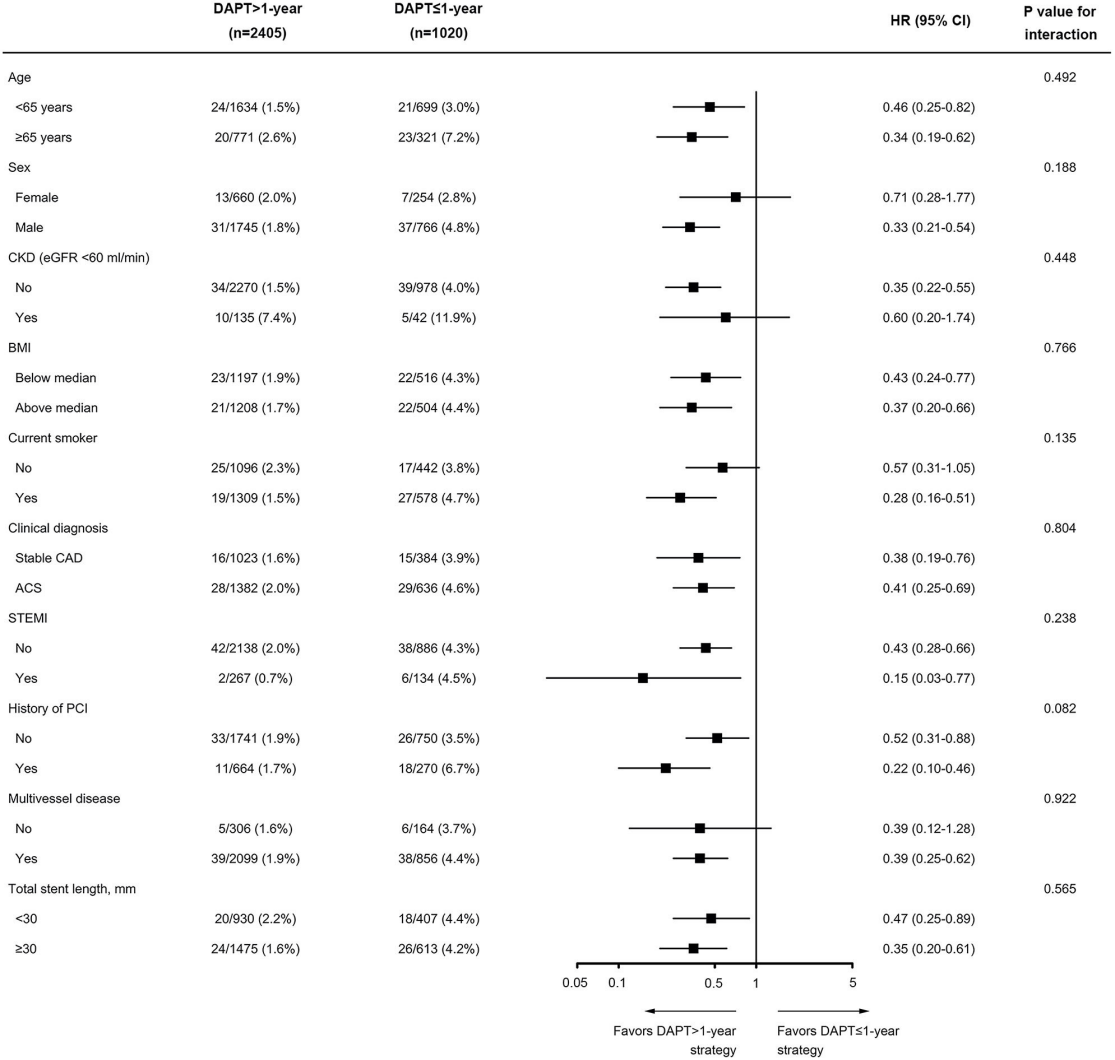
Subgroup analysis of the primary efficacy endpoint of MACCE according to duration of DAPT (DAPT >1-year vs. DAPT ≤ 1-year). Data are shown as the number of MACCE per total number of patients in that subgroup and the event rate. BMI was calculated as weight in kilograms divided by height in meters squared. The *P*-value for interaction represents the likelihood of interaction between the variable and the treatment. ACS, acute coronary syndrome; BMI, body mass index; CAD, coronary artery disease; CKD, chronic kidney disease; PCI, percutaneous coronary intervention; STEMI, ST-segment elevation myocardial infarction.

The treatment effects of continuing DAPT beyond 1 year compared with DAPT cessation within 1 year after PCI were consistent across various subgroups for the MACCE, including the subgroups according to sex (male vs. female), clinical presentation (ACS vs. stable CAD), and presence or absence of STEMI (Figure 5). When potential interactions with clinically relevant bleeding and net clinical benefit were analyzed, the results were also homogeneous across multiple subgroups, with no evidence of a significant modulation or interaction (Supplementary Figures 2, 3). The overall findings of the primary analysis remained unchanged after excluding patients with STEMI (Supplementary Table 4). In addition, when the data set was restricted to only high-risk diabetic subjects with non-ST-elevation ACS, the estimated HRs of adverse events were similar to the main results (Supplementary Table 5).

## Discussion

This analysis, using a large-scale, prospective, real-world registry, is the first assessment of the effect of extended duration DAPT on clinical outcomes in 3,425 high-risk “TWILIGHT-like” diabetic patients. This study has several principle findings as follows: (1) prolonging DAPT with aspirin and clopidogrel beyond 1 year was associated with fewer MACCE, including cardiovascular death, without a significant difference in clinically relevant bleeding, compared with ≤ 1-year DAPT; (2) The incremental efficacy with acceptable bleeding resulting in a net clinical benefit of prolonged DAPT suggested that long-term (≥1-year) DAPT might be a preferred treatment option for this high-risk diabetic patient population; (3) The advantage of DAPT >1-year with respect to ischemic complications was consistent across after multivariable Cox regression, IPTW adjustment, and a number of high-risk enrichment factors. Collectively, DM-tailored, intensified antiplatelet strategies remain an unmet clinical need to mitigate the long-term atherothrombotic ischemic events.

The appropriate duration of DAPT after PCI with DES remains an important subject of debate (2, 3). The existing evidence suggested that a shorter period (3 to 6 months) of DAPT post-PCI might suffice the need to reduce bleedings and prevent largely local (i.e., stent-related) complications in a low-risk population (9, 22). After an initially recommended period, however, identifying certain high-risk patients who may derive additional benefit from prolonging DAPT for the prevention of increasingly systemic thrombotic events becomes the key. A large body of evidence has described that treatment with DAPT over 12 months enabled reduction of the risk of either ST or atherothrombotic events related to an area remote from the stent (4, 5, 23, 24). However, the unavoidable corollary to long-term DAPT after DES placement is a safety concern with regards to the increased risk of bleeding, a complication that is a strong and independent correlate of post-PCI mortality risk (25). In this scenario, the risk/benefit ratio between stopping or continuing DAPT requires clinicians to adopt a more nuanced and individualized approach that incorporates patient's inherent risks for both recurrent bleeding and thrombosis. Of note, participants in the TWILIGHT trial were enriched with both clinical and procedural high-risk criteria who were at an elevated risk for bleeding or ischemic events post-PCI (10, 11). Considering that DM is a key clinical inclusion criterion in “TWILIGHT-like” patients (11), and the well-established relationships of DM with accelerated atherosclerosis and high-risk anatomic characteristics (13, 14), the benefits of longer duration DAPT vs. short duration DAPT in diabetic patients with concomitant high-risk angiographic feature warrants investigation. To the best of our knowledge, the optimal duration of DAPT after PCI for high-risk diabetic patients in real-world treatment practice is uncertain. Therefore, we compared long-term clinical outcomes after PCI between DAPT >1-year and DAPT ≤ 1-year among event-free patients with DM undergoing high-risk PCI after the first year.

It is well known that DM is a widely recognized risk factor for poorer outcomes after PCI (12, 26). With respect to DAPT strategies in diabetic patients, whether DM should be taken into consideration in the choice of the optimal course of DAPT remains a fundamental concern. Recently, it was speculated that the presence of DM could be an important determinant for who benefited from the extended DAPT due to the increased related ischemic risk (3, 27). Further, DM has been recognized as an independent variable in DAPT score, the presence of which may inform extension of DAPT beyond 1 year (28). Indeed, two large observational studies have suggested that long-term (>12-month) clopidogrel treatment with aspirin resulted in reduction of very late all-cause death or MI in diabetic patients receiving DES (27, 29). The OPT-CAD study evaluating the optimal antiplatelet therapy for Chinese patients with coronary artery disease reported that premature discontinuation of DAPT before 12 months experienced significantly increased rates of patient-oriented composite endpoints in high-risk diabetic patients (30). Nevertheless, observations from previous randomized controlled trials of DAPT duration noted conflicting findings with respect to the relative benefit-risk profile observed in patients with DM. In the EXCELLENT trial, there was a significant reduction in target vessel failure with longer DAPT duration among diabetic patients (31). Similarly, the sub-analysis of the DAPT trial provided further evidence in support of the concept that extended thienopyridine over 12 months after coronary stenting was associated with lower rates of MI among patients with DM (32). In contrast, neither PRODIGY, SECURITY, nor I-LOVE-IT 2 trials showed a differential DAPT duration benefit for ischemic events reduction in patients with DM (33–35). In particular, an analysis from a pooled dataset of six randomized trials (*N* = 11,473) also demonstrated that the efficacy of longer duration in reduction of ischemic events was comparable with that of short-term (≤ 6 months) DAPT, but a higher rate of bleeding events among patients with DM (36).

It should be emphasized, however, that previous studies evaluating the efficacy and safety of a longer period (>12 months) of DAPT in patients with DM undergoing PCI mainly enrolled populations of all-comers or low-ischemic risk. However, our study represented high-risk DM patients who are also required to have a high-risk angiographic feature (multivessel CAD, thrombotic lesions, total stent length >30 mm, LM/proximal LAD lesion, bifurcation lesions with two-stent strategy, and calcified target lesions using debulking devices), thus the high-risk nature of cohort portends an increased risk of thrombotic events. There are some possible mechanistic explanations for a greater benefit of long-term DAPT in the context of high-risk DM patient population. First, the higher prevalence of reduced sensitivity to aspirin among patients with DM, which, in turn, is one of the pathophysiological causes of increased platelet reactivity and contributes to the enhanced thrombotic risk of this patient population, indicating that aspirin monotherapy (<1-year DAPT) may be inadequate to prevent major adverse cardiac events in diabetic patients (37). On the other hand, the manifestation of a state of inadequate levels of sustained platelet inhibition with aspirin regimens due to high platelet turnover rates from patients with DM potentially limits the ischemic benefits of low-dose aspirin (38). Second, diabetic patients in our study had worse baseline risk, characterizing higher rates of hypertension (69.8%), smoking (55.1%), dyslipidemia (72.6%), prior revascularization (PCI or CABG) (29.4%), and prior stroke (13.0%), an effect that might allow for extended DAPT to show a greater magnitude of treatment effect. Additionally, the higher proportions of multivessel disease (86.3%), ACC/AHA type B2/C lesions (80.4%), total stent length <30 mm (63.8%), and an obstructive LM or proximal LAD lesion (42.5%) of the diabetic cohort may predispose individuals to benefit more from continuing DAPT over the mandatory period after DES implantation, an important finding given that the presence of DM further increases future risk of atherothrombotic events in a synergistic fashion in patients with multivessel CAD and complex lesions (39–41). Third, in the PROSPECT trial, DM was considered as a powerful predictor of major adverse cardiovascular events related to non-culprit lesions (NCLs) compared with patients without DM, despite frequently mild on angiographic assessment (42). Overall, these observations indicate that prolonged DAPT improves cardiovascular outcomes in high-risk diabetic patients, probably by reducing *de novo* atherothrombotic ischemic events.

In contrast to prior evidence from RCTs indicating an increased bleeding risk in patients undergoing long-term DAPT, the present analysis showed that a prolonged duration of DAPT prevented ischemic events with no significant adverse effect on clinically relevant bleeding, which was consistent with the previous studies reported in real-world population (27, 29, 30, 43). This disparity might be explained in part by heterogeneities in study inclusion/exclusion criteria, patient characteristics, clinical practice and pattern, type of antiplatelet therapy, and confounding factors. First, the present study comprised high-risk diabetic patients who were at a higher risk of cardiovascular events after PCI and low risk of bleeding, potentially necessitating a concept of prolonged-duration antiplatelet regimen in such high-risk patients. Indeed, risk factors for high bleeding risk constituted a small portion of the population in this analysis, of which 232 (6.8%) had advanced age (≥75 years), 177 (5.2%) presented CKD, 127 (3.7%) reported anemia (hemoglobin level <12 g/dl in males and <11 g/dl in females), 38 (1.1%) had moderate or severe thrombocytopenia (platelet count <100 × 10^9^/L), and 21 (0.6%) reported previous major bleeding events. Furthermore, there is a differential propensity for ischemic and bleeding risks in response to P2Y_12_ inhibitors between East Asian and Western patients (44). Previous data suggested that in comparison with clopidogrel, both ticagrelor and prasugrel use significantly increased the rate of bleeding events without a clear benefit with regard to ischemic events in East Asian patients (45–47). Of note, considering that potent P2Y_12_ receptor blockers such as ticagrelor or prasugrel were not available in China during the time of recruitment, all of our participants received clopidogrel as the P2Y_12_ inhibitor, which might be the plausible reason for no relative increase in bleeding risk from prolonged DAPT in the current study. Additionally, we excluded high-risk diabetic patients with bleeding complications in the first 12 months after PCI, which likely selected patients at low risk of bleeding after 12 months in our study. It was possible that patients with increased bleeding risk were switched from DAPT to antiplatelet monotherapy beyond 1 year, whereas high-risk patients with diabetes at lower bleeding risk at 1 year were continued on DAPT beyond 1 year, leading to the similar bleeding risk between the two groups.

There is recognition that patients with diabetes exhibit a diminished platelet inhibitory responsiveness to clopidogrel than non-diabetic subjects, which may explain the increased platelet reactivity and prothrombotic milieu that characterize the post-PCI high ischemic risk state in diabetic patients (48, 49). In our present analysis, we did not have data on platelet function testing (PFT) that enabled physicians to contemplate personalized antiplatelet therapy approaches. With respect to DAPT regimens in diabetic patients, limited data are available regarding using PFT for optimizing on-treatment platelet reactivity among patients with diabetes undergoing PCI. The pre-specified analysis from the TROPICAL-ACS trial showed that diabetic patients had markedly higher on-treatment platelet reactivity levels compared with non-diabetic patients in both on clopidogrel and on prasugrel treatment groups (50). The PFT-guided DAPT de-escalation from prasugrel to clopidogrel strategy and a uniform prasugrel treatment strategy showed comparable efficacy in terms of reductions of combined ischemic events (cardiovascular death, MI, stroke) in diabetic patients. Nevertheless, further investigations are certainly warranted to assess the efficacy and safety of PFT-guided DAPT treatment compared with the standard DAPT therapy regimen in patients with diabetes in the setting of real-world, complex practice. Given that there is still no universally agreed and ideal measure of platelet activation for testing antiplatelet efficacy, robust evidence originating from different studies recently suggest that circulating microRNAs (miRNAs) emerge as novel biomarkers for platelet activation (51, 52). Platelet-related microRNA (such as miR-223 and miR-126) levels are able to reflect measurable changes in platelet activation and aggregation after antiplatelet treatment and are predictive of cardiovascular events (52, 53). In this regard, future studies will need to determine whether tailoring the choice and duration of antiplatelet therapies based on platelet miRNA levels might improve patient outcomes in high-risk diabetic patients on DAPT.

The present study has several limitations. First, the Fuwai PCI study is a large, prospective, observational registry study and has the inherent limitations of observational data sets. Although there were robust statistical adjustments using multivariable Cox regression and IPTW analysis, we cannot exclude unmeasured variables as potential confounders in this study. In general, the current results should be considered hypothesis generating and warrant confirmation in dedicated, prospective designed studies. Second, the duration of DAPT was not randomly assigned but was based on attending physicians' decision according to clinical judgment. Although we rigorously adjusted for differences in baseline characteristics to overcome the potential bias that can influence the study outcome using multivariable Cox models and IPTW analysis, unmeasured confounders may have affected our results. Third, since the lower-than-expected rate of individual endpoint could have limited the power of our analyses, our findings may play a chance and cannot draw definitive conclusions. Fourth, information on glycemic control and DM treatment during follow-up was unavailable in our dataset, which might have an impact on the relative efficacy of prolonged DAPT on clinical outcomes after PCI in patients with DM. Moreover, given that newer P2Y_12_ inhibitors (prasugrel and ticagrelor) were not available in China during the time of this registry, our population received clopidogrel as the P2Y_12_ inhibitor; accordingly, whether our results apply to the potent P2Y_12_ agents remains to be established. Last, it is known that the East Asian population has a higher prevalence of CYP2C19 loss-of-function alleles compared with white individuals, thus contributing to a higher level of platelet reactivity in response to clopidogrel treatment in Asians. Unfortunately, we did not have data on PFT or genetic testing that enabled physicians to contemplate personalized antiplatelet therapy approaches. However, neither routine platelet function testing nor genetic testing is recommended for adjusting antiplatelet therapy before or after elective stenting in the 2017 ESC update on DAPT guidelines.

## Conclusions

In this real-world retrospective analysis of diabetic patients with concomitant high-risk angiographic features who were event-free at 1 year after PCI, extended-term (>1-year) DAPT with clopidogrel and aspirin reduced the risk of MACCE and mortality compared with DAPT discontinuation within 1 year without significant differences in terms of clinically relevant bleeding. As such, long-term DAPT may be considered when contemplating an antiplatelet therapy in high-risk PCI patients with DM for a broader coronary atherothrombotic event protection.

## Data Availability Statement

The raw data supporting the conclusions of this article will be made available by the authors, without undue reservation.

## Ethics Statement

The studies involving human participants were reviewed and approved by the hospital's ethical review board (Fuwai Hospital, Beijing, China). The patients/participants provided their written informed consent to participate in this study.

## Author Contributions

H-YW and K-FD contributed to the study design and interpretation of the results. H-YW, Z-XC, DY, W-HS, LF, R-LG, and Y-JY collected the data. H-YW and Z-XC analyzed the data. H-YW and K-FD prepared the manuscript. H-YW, R-LG, Y-JY, and K-FD revised the manuscript. All authors contributed to the article and approved the submitted version.

## Conflict of Interest

The authors declare that the research was conducted in the absence of any commercial or financial relationships that could be construed as a potential conflict of interest.
