# Supplementary material for: Optimal Strategy for Antiplatelet Therapy After Coronary Drug-Eluting Stent Implantation in High-Risk “TWILIGHT-like” Patients With Diabetes Mellitus
Source: Front Cardiovasc Med. 2020 Nov 27;7:586491. doi: 10.3389/fcvm.2020.586491 (PMC7728996; doi:10.3389/fcvm.2020.586491)
Supplement: Supplementary file 1 [file Table_1.DOCX]

**SUPPLEMENTAL MATERIAL**

**Title:** Optimal strategy for antiplatelet therapy after coronary drug-eluting stent implantation in high-risk "TWILIGHT-like" patients with diabetes mellitus

**Methods**

**PCI procedures**

Unfractionated heparin was used for anticoagulation during the procedure to achieve an activated clotting time of 250 to 300 seconds. Treatment strategy for access site, revascularization treatment strategy, use of glycoprotein IIb/IIIa inhibitor, and use of intravascular imaging assessment were all carried out at the operators’ discretion. The length and diameter of the stent were not restricted. All patients received 300 mg of aspirin orally and a 300 mg or 600 mg clopidogrel loading dose orally before PCI, unless they had previously received these antiplatelet medications. After the procedure, aspirin (100 mg orally once daily) was used indefinitely and clopidogrel (75 mg orally once daily) was maintained for at least 12 months. New P2Y_12_ receptor inhibitors such as prasugrel and ticagrelor were not available during the study period in China. After the procedure, all patients were recommended to receive optimal pharmacological therapy, including statins, β-blockers, or renin-angiotensin system blockade, if indicated, following clinical guidelines (1,2). Patients who discontinued antiplatelet therapy as a result of clinically significant active bleeding or for other procedures were monitored carefully for cardiac events.

**Statistical Analysis**

In order to adjust for possible confounders as much as possible, the inverse probability of treatment weighting (ITPW) Cox proportional hazard regression was performed. The propensity score (PS) has been developed using a nonparsimonious multivariable logistic regression model and considering DAPT duration (DAPT>1-year versus DAPT≤1-year) as dependent variable. IPTW techniques involves assigning each patient a weight (1−p)/ (1−PS) if a control, or weight p/PS if a treated patient, where p is the probability of treatment without any covariate and PS is the value of the PS for that patient. The propensity score include following variables: age, sex, body mass index, current smoking, hypertension, hyperlipidemia, chronic kidney disease, left ventricular ejection fraction, peripheral artery disease, prior myocardial infarction, prior percutaneous coronary intervention, prior coronary artery bypass grafting, prior stroke, acute coronary syndrome, hemoglobin, white blood cell count, platelet count, multivessel disease, target vessel location, in-stent restenosis, heavy calciﬁcation, thrombotic lesion, bifurcation lesions, chronic total occlusion, ACC/ACC type B2/C lesions, SYNTAX score, total vessels treated, total lesions treated, total lesion length, total stents numbers, total stent length, mean stent diameter, type of drug-eluting stents, transradial approach, intravascular ultrasound, and glycoprotein IIb/IIIa use. Balance between the 2 groups after IPTW adjustment was assessed by percent standardized mean differences of all covariates. Variables were considered as balanced between comparative groups after IPTW adjustment if the standardized mean difference of each variable was <10%.

**References:**

1. Valgimigli M, Bueno H, Byrne RA et al. 2017 ESC focused update on dual antiplatelet therapy in coronary artery disease developed in collaboration with EACTS: The Task Force for dual antiplatelet therapy in coronary artery disease of the European Society of Cardiology (ESC) and of the European Association for Cardio-Thoracic Surgery (EACTS). European heart journal 2018;39:213-260.

2. Levine GN, Bates ER, Bittl JA et al. 2016 ACC/AHA Guideline Focused Update on Duration of Dual Antiplatelet Therapy in Patients With Coronary Artery Disease: A Report of the American College of Cardiology/American Heart Association Task Force on Clinical Practice Guidelines. Journal of the American College of Cardiology 2016;68:1082-115.

Supplementary Table 1: Percent standardized differences of variables among unadjusted and IPTW-adjusted cohort

|  | Standardized mean difference (%) | |
| --- | --- | --- |
|  | Unadjusted (N=3425) | IPTW-adjusted (N=3425) |
| Age, yrs | -2.4 | 0.2 |
| Male | 5.8 | 0.2 |
| Body mass index, kg/m^2^ | -1.3 | 0.4 |
| Hypertension | -3.1 | 0.1 |
| Hyperlipidemia | -5.5 | -0.2 |
| Chronic kidney disease | -7.0 | 1.6 |
| Current smoker | 4.5 | -0.2 |
| Insulin | -2.9 | 0.6 |
| PAD | -8.9 | 0.9 |
| Prior MI | -6.4 | 0.3 |
| Prior PCI | -2.6 | 0.3 |
| Prior CABG | -0.9 | 0.0 |
| Prior stroke | 4.0 | -0.4 |
| LVEF, % | 4.2 | 0.2 |
| ACS | 10.0 | 0.2 |
| UA/NSTEMI | 5.7 | -0.4 |
| STEMI | 6.2 | 0.9 |
| Hemoglobin, g/dL | 1.2 | 0.3 |
| Platelet count, 10^9^/L | 2.6 | 0.3 |
| White blood cell count, 10^9^/L | 0.5 | 0.7 |
| Multivessel disease | -9.6 | 0.1 |
| Target vessel |  |  |
| Left main | -4.3 | 0.0 |
| Left anterior descending artery | -1.8 | 0.0 |
| Left circumflex artery | -1.7 | 0.3 |
| Right coronary artery | 0.1 | -0.4 |
| Bypass graft | -2.7 | 0.3 |
| Total lesion length, mm | -6.5 | 1.0 |
| Number of vessels treated | -2.7 | 0.1 |
| Number of lesions treated | -7.3 | 0.3 |
| 1 |  |  |
| 2 |  |  |
| ≥3 |  |  |
| Number of stents implanted | -5.6 | 0.4 |
| Total stent length, mm | -5.5 | 0.8 |
| Mean stent diameter, mm | 3.7 | 0.1 |
| Target lesion morphology |  |  |
| Bifurcation | -4.7 | 0.0 |
| Chronic total occlusion | -5.0 | 0.4 |
| In-stent restenosis | -2.1 | 0.3 |
| Severe calcification | -3.7 | 0.8 |
| Thrombotic lesion | 3.1 | 0.3 |
| Type B2 or C lesion | -2.0 | 0.5 |
| SYNTAX score | -4.4 | 0.1 |
| Type of DES implanted | 0.1 | 0.3 |
| First-generation DES |  |  |
| Second-generation DES |  |  |
| Vascular access site | 2.7 | 0.6 |
| Radial approach |  |  |
| Femoral approach |  |  |
| Use of intravascular ultrasound | -1.7 | 0.5 |
| Glycoprotein IIb/IIIa use | 4.9 | 0.3 |

ACS indicates acute coronary syndrome; CABG, coronary artery bypass grafting; DAPT, dual antiplatelet therapy; LVEF, left ventricular ejection fraction; MI, myocardial infarction; NSTEMI, non-ST-segment elevation myocardial infarction; PAD, peripheral artery disease; PCI, percutaneous coronary intervention; PARIS, Patterns of Non-Adherence to Anti-Platelet Regimen in Stented Patients; STEMI, ST-segment elevation myocardial infarction; SYNTAX, Synergy Between PCI With Taxus and Cardiac Surgery; and UA, unstable angina.

Supplementary Table 2: Baseline clinical characteristics in high-risk patients with diabetes mellitus according to DAPT duration stratified by sex

|  | Female (n=914) | | | Male (n=2511) | | |
| --- | --- | --- | --- | --- | --- | --- |
|  | DAPT≤1-year  (n=254) | DAPT>1-year  (n=660) | P value | DAPT≤1-year  (n=766) | DAPT>1-year  (n=1745) | P value |
| Age, years | 64.26 ± 8.09 | 64.26 ± 7.90 | 0.990 | 57.85 ± 9.74 | 57.95 ± 10.00 | 0.812 |
| Body mass index, kg/m^2^ | 25.80 ± 3.36 | 25.89 ± 3.25 | 0.713 | 26.41 ± 3.13 | 26.45 ± 3.11 | 0.750 |
| Hypertension | 196 (77.2) | 523 (79.2) | 0.492 | 506 (66.1) | 1166 (66.8) | 0.709 |
| Hyperlipidemia | 182 (71.7) | 469 (71.1) | 0.859 | 541 (70.6) | 1295 (74.2) | 0.062 |
| Chronic kidney disease | 16 (6.3) | 60 (9.1) | 0.171 | 26 (3.4) | 75 (4.3) | 0.289 |
| Current smoker | 22 (8.7) | 66 (10.0) | 0.539 | 556 (72.6) | 1243 (71.2) | 0.489 |
| PAD | 6 (2.4) | 26 (3.9) | 0.245 | 19 (2.5) | 71 (4.1) | 0.049 |
| Prior MI | 30 (11.8) | 98 (14.8) | 0.236 | 179 (23.4) | 458 (26.2) | 0.127 |
| Prior PCI | 56 (22.0) | 152 (23.0) | 0.751 | 214 (27.9) | 512 (29.3) | 0.475 |
| Prior CABG | 17 (6.7) | 33 (5.0) | 0.313 | 36 (4.7) | 97 (5.6) | 0.376 |
| Prior stroke | 41 (16.1) | 100 (15.2) | 0.710 | 101 (13.2) | 202 (11.6) | 0.254 |
| LVEF, % | 63.53 ± 7.42 | 63.74 ± 6.65 | 0.682 | 62.33 ± 7.30 | 61.78 ± 7.80 | 0.097 |
| Indication for PCI |  |  | 0.014 |  |  | 0.111 |
| Stable CAD | 93 (36.6) | 301 (45.6) |  | 291 (38.0) | 722 (41.4) |  |
| ACS | 161 (63.4) | 359 (54.4) |  | 475 (62.0) | 1023 (58.6) |  |
| UA/NSTEMI | 144 (56.7) | 314 (47.6) | 0.014 | 358 (46.7) | 801 (45.9) | 0.700 |
| STEMI | 17 (6.7) | 45 (6.8) | 0.946 | 117 (15.3) | 222 (12.7) | 0.085 |
| Hemoglobin, g/dL | 12.93 ± 1.26 | 12.88 ± 1.26 | 0.604 | 14.63 ± 1.36 | 14.68 ± 1.39 | 0.386 |
| Platelet count, 10^3^/dL | 224.59 ± 61.01 | 219.59 ± 57.36 | 0.246 | 199.66 ± 54.32 | 198.62 ± 54.37 | 0.659 |
| White blood cell count, 10^3^/mL | 6.60 ± 1.52 | 6.57 ± 1.57 | 0.783 | 6.95 ± 1.65 | 6.96 ± 1.72 | 0.867 |
| HbA1c, % | 7.62 ± 1.38 | 7.65 ± 1.34 | 0.799 | 7.45 ± 1.30 | 7.66 ± 1.34 | <0.001 |
| PARIS thrombotic risk score | 3.15 ± 1.92 | 3.01 ± 1.95 | 0.330 | 3.38 ± 1.66 | 3.46 ± 1.79 | 0.331 |
| PARIS bleeding risk score | 3.76 ± 2.26 | 3.90 ± 2.28 | 0.406 | 3.65 ± 1.84 | 3.75 ± 2.00 | 0.237 |
| PRECISE-DAPT score | 17.67 ± 9.52 | 17.58 ± 8.71 | 0.888 | 8.99 ± 7.16 | 9.30 ± 7.46 | 0.322 |
| DAPT score | 1.39 ± 1.17 | 1.37 ± 1.22 | 0.828 | 2.40 ± 1.21 | 2.42 ± 1.21 | 0.698 |
| Medication |  |  |  |  |  |  |
| Aspirin | 252 (99.2) | 655 (99.2) | 0.963 | 755 (98.6) | 1724 (98.8) | 0.632 |
| Clopidogrel | 252 (99.2) | 654 (99.1) | 0.860 | 757 (98.8) | 1719 (98.5) | 0.535 |
| Beta-blocker | 233 (91.7) | 602 (91.2) | 0.802 | 704 (91.9) | 1620 (92.8) | 0.413 |
| Calcium channel blockers | 153 (60.2) | 389 (58.9) | 0.721 | 367 (47.9) | 831 (47.6) | 0.894 |
| Statin | 237 (93.3) | 625 (94.7) | 0.416 | 725 (94.6) | 1684 (96.5) | 0.030 |
| Antidiabetic drugs at baseline |  |  |  |  |  |  |
| OADs | 95 (37.4) | 250 (37.9) | 0.894 | 348 (45.4) | 726 (41.6) | 0.074 |
| Insulin | 67 (26.4) | 154 (23.3) | 0.336 | 180 (23.5) | 459 (26.3) | 0.137 |

Values are n (%) or mean ± SD. ACEI indicates angiotensin-converting enzyme inhibitors; ACS, acute coronary syndrome; ARB, angiotensin receptor blockers; CABG, coronary artery bypass grafting; DAPT, dual antiplatelet therapy; HbA1c, glycosylated hemoglobin; LVEF, left ventricular ejection fraction; MI, myocardial infarction; NSTEMI, non-ST-segment elevation myocardial infarction; OADs, oral antidiabetic drugs; PCI, percutaneous coronary intervention; STEMI, ST-segment elevation myocardial infarction; and UA, unstable angina.

Supplementary Table 3: Procedural characteristics in high-risk patients with diabetes mellitus according to DAPT duration stratified by sex

|  | Female (n=914) | | | Male (n=2511) | | |
| --- | --- | --- | --- | --- | --- | --- |
|  | DAPT≤1-year  (n=254) | DAPT>1-year  (n=660) | P value | DAPT≤1-year  (n=766) | DAPT>1-year  (n=1745) | P value |
| Multivessel CAD | 211 (83.1) | 590 (89.4) | 0.009 | 645 (84.2) | 1509 (86.5) | 0.133 |
| Target vessel |  |  |  |  |  |  |
| Left anterior descending artery | 229 (90.2) | 596 (90.3) | 0.947 | 669 (87.3) | 1535 (88.0) | 0.658 |
| Left circumflex artery | 44 (17.3) | 114 (17.3) | 0.986 | 160 (20.9) | 383 (21.9) | 0.552 |
| Right coronary artery | 56 (22.0) | 131 (19.8) | 0.460 | 165 (21.5) | 391 (22.4) | 0.630 |
| Left main coronary artery | 9 (3.5) | 18 (2.7) | 0.514 | 19 (2.5) | 66 (3.8) | 0.097 |
| Bypass graft | 0 (0.0) | 1 (0.2) | 1.000 | 2 (0.3) | 7 (0.4) | 0.731 |
| Total lesion length, mm | 41.11 ± 28.03 | 40.49 ± 26.05 | 0.752 | 40.25 ± 25.44 | 42.88 ± 28.43 | 0.027 |
| Number of vessels treated | 1.30 ± 0.51 | 1.28 ± 0.50 | 0.598 | 1.30 ± 0.50 | 1.33 ± 0.53 | 0.235 |
| Number of lesions treated |  |  | 0.793 |  |  | 0.068 |
| 1 | 162 (63.8) | 436 (66.1) |  | 472 (61.6) | 1063 (60.9) |  |
| 2 | 73 (28.7) | 180 (27.3) |  | 239 (31.2) | 508 (29.1) |  |
| ≥3 | 19 (7.5) | 44 (6.7) |  | 55 (24.0) | 174 (10.0) |  |
| Number of stents implanted | 2.04 ± 1.14 | 1.99 ± 1.04 | 0.507 | 1.98 ± 1.00 | 2.09 ± 1.17 | 0.035 |
| ≥3 stents implanted | 69 (27.2) | 169 (25.6) | 0.630 | 193 (25.2) | 486 (27.9) | 0.168 |
| Total stent length, mm | 44.71 ± 27.11 | 44.39 ± 26.71 | 0.870 | 44.37 ± 25.64 | 46.53 ± 29.05 | 0.076 |
| Total stent length>30 mm | 162 (63.8) | 414 (62.7) | 0.768 | 490 (64.0) | 1118 (64.1) | 0.962 |
| Mean stent diameter, mm | 2.85 ± 0.51 | 2.87 ± 0.52 | 0.603 | 3.02 ± 0.54 | 2.99 ± 0.55 | 0.215 |
| Target lesion morphology |  |  |  |  |  |  |
| Bifurcation | 29 (11.4) | 95 (14.4) | 0.239 | 123 (16.1) | 305 (17.5) | 0.383 |
| Chronic total occlusion | 10 (3.9) | 41 (6.2) | 0.179 | 68 (8.9) | 176 (10.1) | 0.346 |
| In-stent restenosis | 8 (3.1) | 21 (3.2) | 0.980 | 44 (5.7) | 113 (6.5) | 0.486 |
| Severe calcification | 10 (3.9) | 31 (4.7) | 0.619 | 24 (3.1) | 66 (3.8) | 0.420 |
| Thrombotic lesion | 13 (5.1) | 15 (2.3) | 0.025 | 31 (4.0) | 74 (4.2) | 0.823 |
| Type B2 or C lesion | 206 (81.1) | 536 (81.2) | 0.970 | 608 (79.4) | 1402 (80.3) | 0.575 |
| SYNTAX score | 11.99 ± 8.11 | 12.47 ± 8.03 | 0.420 | 12.20 ± 8.18 | 12.52 ± 8.20 | 0.369 |
| Vascular access site |  |  | 0.537 |  |  | 0.230 |
| Radial approach | 221 (87.0) | 584 (88.5) |  | 707 (92.3) | 1585 (90.8) |  |
| Femoral approach | 33 (13.0) | 76 (11.5) |  | 59 (7.7) | 160 (9.2) |  |
| Intravascular ultrasound use | 12 (4.7) | 22 (3.3) | 0.320 | 43 (5.6) | 117 (6.7) | 0.303 |
| Glycoprotein IIb/IIIa use | 36 (14.2) | 88 (13.3) | 0.740 | 141 (18.4) | 286 (16.4) | 0.215 |
| DES type |  |  | 0.751 |  |  | 0.911 |
| DES, ﬁrst-generation | 24 (9.4) | 67 (10.2) |  | 85 (11.1) | 191 (10.9) |  |
| DES, second-generation | 230 (90.6) | 593 (89.8) |  | 681 (88.9) | 1554 (89.1) |  |

Values are n (%) or mean ± SD. CAD indicates coronary artery disease; DES, drug-eluting stent.

Supplementary Table 4: Adverse clinical events in high-risk diabetic patients according to DAPT duration after excluding patients with STEMI (n=3024)

|  | DAPT>1-year (n=2138) | DAPT≤1-year (n=886) | Univariate Analysis | | Multivariable Analysis* | |
| --- | --- | --- | --- | --- | --- | --- |
|  |  |  | HR (95% CI) | P value | Adjusted HR (95% CI) | P value |
| Major adverse cardiac and cerebrovascular events | 42 (2.0%) | 38 (4.3%) | 0.427 (0.275-0.663) | <0.001 | 0.402 (0.258-0.625) | <0.001 |
| CV death, myocardial infarction, or ischemic stroke | 37 (1.7%) | 29 (3.3%) | 0.494 (0.304-0.805) | 0.005 | 0.462 (0.283-0.753) | 0.002 |
| All-cause death | 4 (0.2%) | 26 (2.9%) | 0.060 (0.021-0.171) | <0.001 | 0.056 (0.020-0.162) | <0.001 |
| CV death | 3 (0.1%) | 15 (1.7%) | 0.078 (0.023-0.271) | <0.001 | 0.071 (0.020-0.248) | <0.001 |
| Myocardial infarction | 13 (0.6%) | 5 (0.6%) | 1.005 (0.358-2.822) | 0.993 | 0.937 (0.332-2.645) | 0.901 |
| Stroke | 27 (1.3%) | 14 (1.6%) | 0.743 (0.389-1.420) | 0.369 | 0.710 (0.371-1.362) | 0.303 |
| Ischemic stroke | 23 (1.1%) | 14 (1.6%) | 0.637 (0.327-1.241) | 0.185 | 0.608 (0.311-1.190) | 0.146 |
| Definite/probable stent thrombosis | 5 (0.2%) | 5 (0.6%) | 0.385 (0.111-1.336) | 0.133 | 0.341 (0.096-1.206) | 0.095 |
| Clinically relevant bleeding | 24 (1.1%) | 9 (1.0%) | 1.009 (0.468-2.176) | 0.982 | 1.127 (0.505-2.517) | 0.770 |
| Net clinical benefit | 63 (2.9%) | 47 (5.3%) | 0.514 (0.352-0.913) | 0.001 | 0.492 (0.335-0.722) | <0.001 |

Data presented as number of events (%). *The candidate covariates considered for inclusion in the model for ischemic outcomes were age, sex, current smoker, hypertension, chronic kidney disease, left ventricular ejection fraction, peripheral artery disease, prior MI, prior PCI or CABG, DES type, multivessel CAD, treated lesion in the left main or left anterior descending artery, total lesion length, and total stents numbers. The candidate covariates considered for inclusion in the model for clinically relevant bleeding were age, sex, body mass index, chronic kidney disease, history of major bleeding, and anemia. The candidate covariates considered for inclusion in the model for net clinical benefit were age, sex, body mass index, current smoker, hypertension, chronic kidney disease, left ventricular ejection fraction, peripheral artery disease, prior MI, prior PCI or CABG, DES type, multivessel CAD, treated lesion in the left main or left anterior descending artery, total lesion length, total stents numbers, history of bleeding, and anemia.

CI, confidence interval; CV, cardiovascular; DAPT, dual antiplatelet therapy; HR, hazard ratio; STEMI, ST-segment elevation myocardial infarction. Other abbreviations as in Supplementary Table 2 and 3.

Supplementary Table 5: Adverse clinical events in high-risk diabetic patients according to DAPT duration and presenting with NSTE-ACS (n=1617)

|  | DAPT>1-year (n=1115) | DAPT≤1-year (n=502) | Univariate Analysis | | Multivariable Analysis* | |
| --- | --- | --- | --- | --- | --- | --- |
|  |  |  | HR (95% CI) | P value | Adjusted HR (95% CI) | P value |
| Major adverse cardiac and cerebrovascular events | 26 (2.3%) | 23 (4.6%) | 0.472 (0.269-0.829) | 0.009 | 0.469 (0.267-0.824) | 0.008 |
| CV death, myocardial infarction, or ischemic stroke | 24 (2.2%) | 18 (3.6%) | 0.558 (0.303-1.031) | 0.062 | 0.554 (0.300-1.025) | 0.060 |
| All-cause death | 3 (0.3%) | 14 (2.8%) | 0.090 (0.026-0.315) | <0.001 | 0.089 (0.025-0.302) | <0.001 |
| CV death | 2 (0.2%) | 8 (1.6%) | 0.109 (0.023-0.514) | 0.005 | 0.111 (0.024-0.524) | 0.005 |
| Myocardial infarction | 6 (0.5%) | 3 (0.6%) | 0.822 (0.205-3.298) | 0.782 | 0.829 (0.206-3.334) | 0.792 |
| Stroke | 19 (1.7%) | 10 (2.0%) | 0.796 (0.369-1.715) | 0.369 | 0.789 (0.364-1.701) | 0.542 |
| Ischemic stroke | 18 (1.6%) | 10 (2.0%) | 0.753 (0.347-1.634) | 0.185 | 0.744 (0.342-1.621) | 0.457 |
| Definite/probable stent thrombosis | 3 (0.3%) | 3 (0.6%) | 0.403 (0.081-2.012) | 0.268 | 0.403 (0.080-2.018) | 0.269 |
| Clinically relevant bleeding | 12 (1.1%) | 6 (1.2%) | 0.839 (0.314-2.240) | 0.725 | 0.948 (0.331-2.718) | 0.921 |
| Net clinical benefit | 37 (3.3%) | 29 (5.8%) | 0.532 (0.326-0.866) | 0.011 | 0.512 (0.312-0.842) | 0.008 |

Data presented as number of events (%). *The candidate covariates considered for inclusion in the model for ischemic outcomes were age, sex, current smoker, prior PCI or CABG, DES type, multivessel CAD, total lesion length, and total stents numbers. The candidate covariates considered for inclusion in the model for clinically relevant bleeding were age, sex, chronic kidney disease, history of major bleeding, and anemia. The candidate covariates considered for inclusion in the model for net clinical benefit were age, sex, current smoker, chronic kidney disease, prior PCI or CABG, DES type, multivessel CAD, total lesion length, total stents numbers, history of bleeding, and anemia.

CI, confidence interval; CV, cardiovascular; DAPT, dual antiplatelet therapy; HR, hazard ratio; NSTE-ACS, non-ST-elevation acute coronary syndrome. Other abbreviations as in Supplementary Table 2 and 3.

Supplementary Figure 1: Univariate and multivariate subdistribution hazards ratios for major cardiovascular and cerebrovascular events (MACCE) according to DAPT duration by Fine-Gray subdistribution hazards models


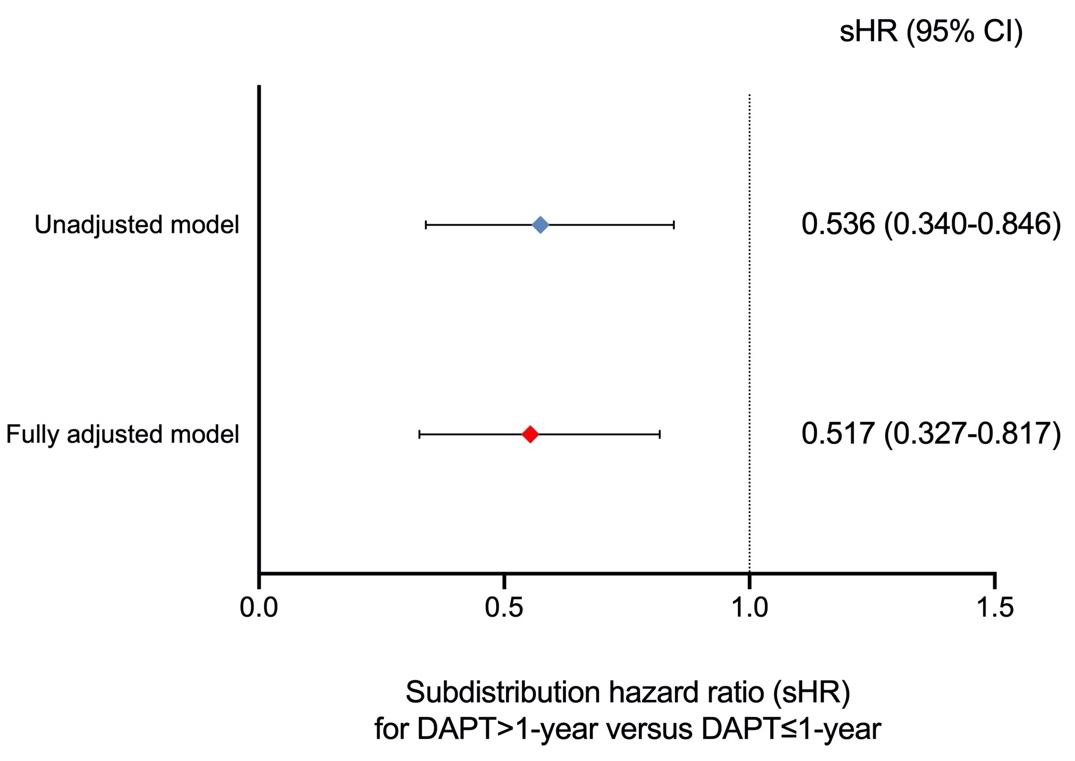


MACCE was defined as a composite of cardiovascular death, myocardial infarction, or stroke. sHR represents the risk of MACCE for DAPT>1-year versus DAPT≤1-year while treating non-cardiovascular death as competing risk. Adjusted sHR account for age, sex, current smoker, hypertension, chronic kidney disease, acute coronary syndrome, left ventricular ejection fraction, peripheral artery disease, prior MI, prior PCI or CABG, DES type, multivessel CAD, treated lesion in the left main or left anterior descending artery, total lesion length, total stents numbers, and the competing risk of non-cardiovascular death.

CI indicates conﬁdence interval; DAPT, dual antiplatelet therapy; HR, hazard ratio; sHR, subdistribution hazards ratios; Other abbreviations as in Supplementary Table 2 and 3.


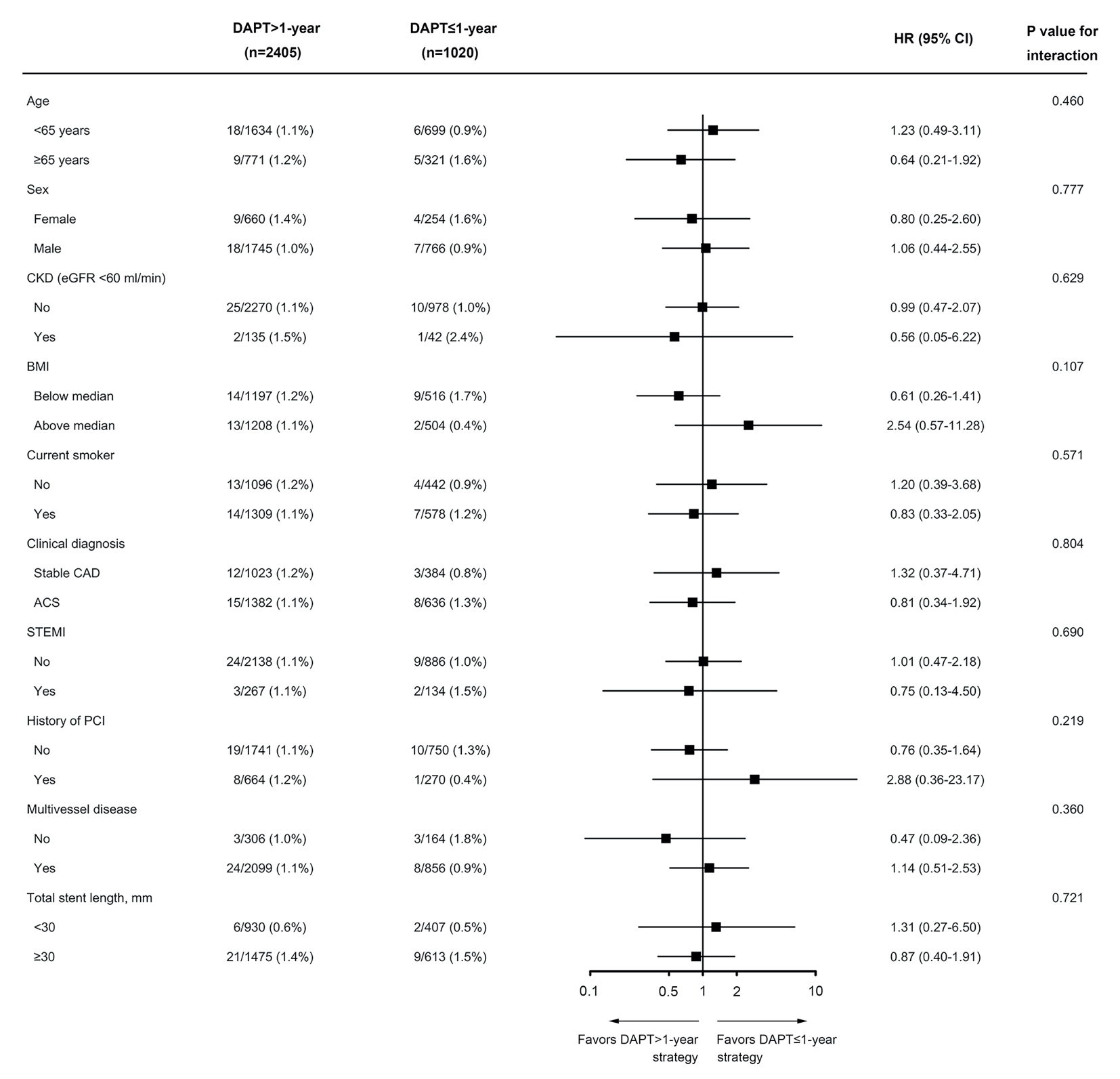
Supplementary Figure 2: Subgroups analysis of clinically relevant bleeding according to duration of DAPT (DAPT>1-year versus DAPT≤1-year)

Data are shown as the number of clinically relevant bleeding per total number of patients in that subgroup and the event rate. BMI was calculated as weight in kilograms divided by height in meters squared. The P value for interaction represents the likelihood of interaction between the variable and the treatment.

ACS = acute coronary syndrome; BMI = body mass index; CAD = coronary artery disease; CKD = chronic kidney disease; PCI, percutaneous coronary intervention; STEMI, ST-segment elevation myocardial infarction.


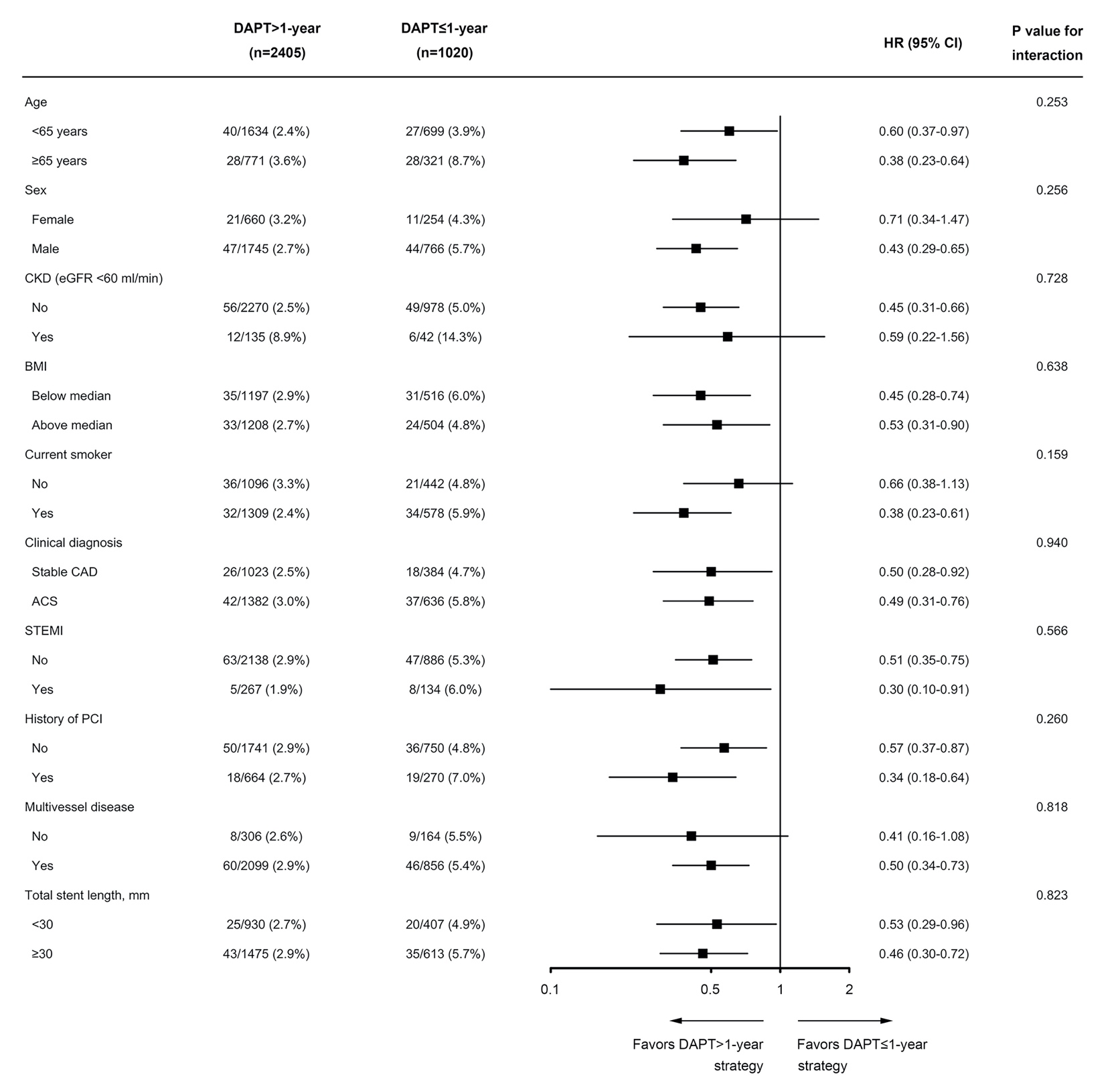
Supplementary Figure 3: Subgroups analysis of net clinical benefit according to duration of DAPT (DAPT>1-year versus DAPT≤1-year)

Data are shown as the number of net clinical benefit per total number of patients in that subgroup and the event rate. BMI was calculated as weight in kilograms divided by height in meters squared. The P value for interaction represents the likelihood of interaction between the variable and the treatment.

ACS = acute coronary syndrome; BMI = body mass index; CAD = coronary artery disease; CKD = chronic kidney disease; PCI, percutaneous coronary intervention; STEMI, ST-segment elevation myocardial infarction.
